# Supplementary material for: Racial disparities in continuous glucose monitoring-based 60-min glucose predictions among people with type 1 diabetes
Source: PLOS Digit Health. 2025 Jun 30;4(6):e0000918. doi: 10.1371/journal.pdig.0000918 (PMC12208448; doi:10.1371/journal.pdig.0000918)
Supplement: S1 Table — Values in the table are given as absolute values (%). (PDF) [file pdig.0000918.s001.pdf]

Table S1. Results from Surveillance Error Grid for White participants. Values in the table are given as absolute values (%)

			Proportion of White participants					
	Model	Zone	0	20	40	60	80	100
White	LOCF	None	69%					
		Slight	26%					
		Moderate	4% … for all ratios					
		High	0%					
		Extreme	0%					
	Base-Individual	None	66%					
		Slight	28%					
		Moderate	6% … for all ratios					
		High	0%					
		Extreme	0%					
	Base-Generalized	None	72%	73%	73%	73%	73%	73%
		Slight	25%	25%	25%	24%	24%	24%
		Moderate	3%	3%	3%	3%	3%	3%
		High	0%	0%	0%	0%	0%	0%
		Extreme	0%	0%	0%	0%	0%	0%
	Transfer Learned	None	72%	72%	73%	73%	73%	73%
		Slight	25%	24%	24%	24%	24%	24%
		Moderate	3%	3%	3%	3%	3%	3%
		High	0%	0%	0%	0%	0%	0%
		Extreme	0%	0%	0%	0%	0%	0%
