# Supplementary material for: Racial disparities in continuous glucose monitoring-based 60-min glucose predictions among people with type 1 diabetes
Source: PLOS Digit Health. 2025 Jun 30;4(6):e0000918. doi: 10.1371/journal.pdig.0000918 (PMC12208448; doi:10.1371/journal.pdig.0000918)
Supplement: S2 Table — Values in the table are given as absolute values (%). (PDF) [file pdig.0000918.s002.pdf]

Table S2. Results from Surveillance Error Grid for Black participants. Values in the table are given as absolute values (%)

			Proportion of White participants					
Black	Model	Zone	0	20	40	60	80	100
	LOCF	None<br>Slight<br>Moderate<br>High<br>Extreme	71%<br>24%<br>4% … for all ratios<br>0%<br>0%					
	Base-Individual	None<br>Slight<br>Moderate<br>High<br>Extreme	66%<br>27%<br>6% … for all ratios<br>0%<br>0%					
	Base-Generalized	None<br>Slight<br>Moderate<br>High<br>Extreme	75%<br>23%<br>3%<br>0%<br>0%	74%<br>23%<br>3%<br>0%<br>0%	75%<br>22%<br>3%<br>0%<br>0%	75%<br>22%<br>3%<br>0%<br>0%	74%<br>22%<br>3%<br>0%<br>0%	75%<br>22%<br>3%<br>0%<br>0%
	Transfer Learned	None<br>Slight<br>Moderate<br>High<br>Extreme	75%<br>22%<br>3%<br>0%<br>0%	74%<br>23%<br>3%<br>0%<br>0%	75%<br>22%<br>3%<br>0%<br>0%	75%<br>22%<br>3%<br>0%<br>0%	74%<br>22%<br>3%<br>0%<br>0%	75%<br>22%<br>3%<br>0%<br>0%
