# Supplementary material for: Racial disparities in continuous glucose monitoring-based 60-min glucose predictions among people with type 1 diabetes
Source: PLOS Digit Health. 2025 Jun 30;4(6):e0000918. doi: 10.1371/journal.pdig.0000918 (PMC12208448; doi:10.1371/journal.pdig.0000918)
Supplement: Attachment — Submitted filename: Response to Reviewers.docx [file pdig.0000918.s004.docx]

Tab 1

Response to Reviewers (PDIG-D-25-00023)

Dear Editors and Reviewers,

We appreciate the constructive feedback on our manuscript. We have revised the manuscript accordingly. Please find our point-by-point responses below including all the revised text. Line numbers indicating the place of changes in the manuscript refer to the version with tracked changes.

Kind regards,

Adam Hulman

on behalf of all co-authors

| Comments and questions | Reviewers |
| --- | --- |
| 1. Does this manuscript meet PLOS Digital Health’s [publication criteria](https://eur01.safelinks.protection.outlook.com/?url=https%3A%2F%2Ftrack.editorialmanager.com%2FCL0%2Fhttps%3A%252F%252Fjournals.plos.org%252Fdigitalhealth%252Fs%252Fjournal-information%2523loc-criteria-for-publication%2F1%2F010f01952af1e395-85a7e531-0b4f-4c96-a56e-8b1da441eaa1-000000%2FCUyyaY6_u0HgPLpDgJj5udbG3LnPqk8dU0156nbgoDg%3D199&data=05%7C02%7Chbt%40ph.au.dk%7Ccd70bb6430b9470467b208dd52d38186%7C61fd1d36fecb47cab7d7d0df0370a198%7C1%7C0%7C638757790541868297%7CUnknown%7CTWFpbGZsb3d8eyJFbXB0eU1hcGkiOnRydWUsIlYiOiIwLjAuMDAwMCIsIlAiOiJXaW4zMiIsIkFOIjoiTWFpbCIsIldUIjoyfQ%3D%3D%7C0%7C%7C%7C&sdata=rVmY%2F3j%2FJY7KQK9LAOhMFx1m%2FUuqGJC3Ljcs1mVRJbo%3D&reserved=0)? Is the manuscript technically sound, and do the data support the conclusions? The manuscript must describe methodologically and ethically rigorous research with conclusions that are appropriately drawn based on the data presented. | Reviewer #1: Partly  Reviewer #2: Partly |
| 2. Has the statistical analysis been performed appropriately and rigorously? | Reviewer #1: I don't know  Reviewer #2: Yes |
| 3. Have the authors made all data underlying the findings in their manuscript fully available (please refer to the Data Availability Statement at the start of the manuscript PDF file)? | Reviewer #1: Yes  Reviewer #2: Yes |
| 4. Is the manuscript presented in an intelligible fashion and written in standard English? | Reviewer #1: Yes  Reviewer #2: Yes |

| Reviewer 1 | Answers |
| --- | --- |
|  | We appreciate the reviewer’s interest and constructive feedback on the manuscript. |
| While interesting, the main result of the study to me seems to be that race does not seem to be a major influence on the error of a blood glucose prediction model. Would the authors agree with that statement?  Now, this could be because of the relatively small sample size or the comparably poor performance of all the models, or other factors, but this finding should be clearly stated and potential caveats discussed. | Thank you for your valuable input regarding our results. Our findings are not as strong as we hypothesized, even though we found a small divergence in performance between the groups. We agree with the reviewer that the sample size played a potential role in this. We have revised the limitations section to pinpoint this:  ‘A limitation of this study is the size of the dataset, **which influenced the statistical power of the analyses**.’ (line 343)  Although the performance of the models is not outstanding, the base-generalized model performs better than the naive prediction and models developed only using data from one individual (~30% lower RMSE). Therefore we do not think that the level of performance could be the reason for not seeing stronger differences between groups, but we cannot exclude that option either.  In general, we acknowledge that the absence of strong evidence should not be interpreted as evidence of absence. We believe that our work is relevant to the field and will inspire further studies to investigate algorithmic fairness in diabetes research and beyond. We have revised the Discussion to elaborate and clarify the points from our response (line 280):  *“We found that the racial composition of training data led to a small statistically significant divergence in performance between the slopes that were fitted to the results from White and Black participants when models were not personalized. This suggests increasing disparity, with performance differences favoring White individuals becoming more pronounced as their proportion in the dataset grows. Although the difference between the performances of the two racial groups might not be large enough to produce clinical inequalities in this study, the findings still indicate that the machine learning models could pick up on racial markers within the CGM data that was used in our study. This stresses the importance of training data composition from the perspective of algorithmic fairness to avoid increasing disparities between ethnic, racial, and demographic groups when considering integrating and using AI-driven healthcare tools. Performance differences disappeared when transfer learning was applied, demonstrating that the imbalance in training data can be mitigated with data from the underrepresented group in the initial training data”* |
| Line 117:  How can datasets created within one participant contain varying proportions of black and white participants? Clarify the sentence. How can datasets created within one participant contain varying proportions of black and white participants? Clarify the sentence.  Line 117: What is meant by “Eleven training sets were created for each participant in the dataset with varying proportions”. | Thank you for highlighting the need for clarification of the study design. The 11 training datasets are created for (within) one participant by the following steps:   1. Select the participant and remove his or her data from the dataset 2. Create 11 training datasets with different proportions of non-Hispanic Black and White participants from the remaining dataset 3. Assign these 11 training datasets to the one participant removed in the first step so that the participant’s data can act as an independent test set 4. Steps 1-3 are repeated for all 205 participants in the dataset. This resulted in 205 participants x 11 training datasets = 2,255 training datasets in total and 205 independent test sets.   The confusing sentences have been revised to clarify the process, and an addition to the figure legend has been made (line 131):  *“For each participant, eleven training sets were created, each including 100 other participants’ data with varying proportions of White and Black participants (0-100%, 10-90%, ..., 100-0%). This resulted in 2,255 training datasets (205 individuals times 11 training sets with varying proportions of race) and 205 independent test sets. By creating 11 subsets of training data for each participant in the dataset, we ensured there was no data leakage from each participant to the 11 training datasets assigned to them.”*  (Figure legend: line 161)  *“*The top panel illustrates progressive training strategies with varying proportions of White and Black patients (from 100%-0% to 0%-100%) marked by the grey rectangles behind the datasets.*”* |
| It seems the authors mainly do within subject model development. While this is interesting, it would be good to see how well the model transfers across participants. Is that possible at all? Is there anything that can be learned from one participant to the next? | Thank you for your thoughtful question. The base-generalized models have been trained on 100 participants each (the number of participants in the smallest group without the test participant). Then the base-generalized models are further trained on individual-level data i.e. tailored to a participant using fine tuning (transfer-learned models, Figure 1). Models trained on data from single individuals are only used as simple benchmarks.  The transfer learned models (i.e. generalized models tailored to individuals) have better performance than both the generalized and the individual models (Table 2). This indicates that transfer-learned models outperform individual models, as they leverage knowledge from other participants, thus demonstrating knowledge transfer across participants.  We have added “fine-tuning” to an arrow in Figure 1 (line 151) and added fine-tuning to the description of the transfer learned model (line 137): *”However, the 11 trained models for each participant were subsequently tailored, i.e., fine-tuned with transfer learning to the given participant’s complete CGM data (except for the last week, which was used for the testing data). (Figure 1)”* |
| Could the authors normalize each RMSE of the transfer or extended model by the respective performance of the base model in exactely that condition? Potentially, this could reveal different effects now covered by the intersubject variability. | Thank you for pointing out the importance of inter-subject variability and its potential impact on the results. We used the linear mixed effects model approach to account for both inter- and intra-subject variability. We are confident that the inclusion of a random intercept is the best statistical approach to handle this. We extended our argument for using this approach in the Methods section (line 185) and now report the variance of the random effect describing inter-individual variability in the dataset, in the Results section (line 236).  Methods:  *“The linear mixed effect model approach was chosen because data points from the same individual cannot be considered independent measurements. A random intercept was included for individuals since it was assumed there would be variation in RMSE results between participants. The variance of the random intercepts describes between-person variability. The model included the following covariates: ratio (proportion of White and Black), race, age, sex, and training data sample size. An interaction term between race and ratio was added to model a potential divergence in performance by race because it was assumed training data composition affected race. This means that a different slope was allowed for the two racial groups when the linear mixed effect models were fitted to the results so the hypothesis could be tested.”*  Results:  “*Between-person variance of RMSE was 0.198 for the Base-Generalized and 0.172 for the Transfer Learned model.*” |
| Is 30 min really the relevant time scale? What about more finely binned data or data at the full native resolution of the measurement device? | While we acknowledge that a higher resolution, i.e. measurements every 5 minutes, would have been desirable, 15-minute time intervals were the highest resolution of the measuring device in the dataset. Although RMSE could have been lower with higher resolution, we do not have an argument for why the relationship between the two groups would have been different in such a scenario. Unfortunately, we are not aware of any other datasets with higher resolution and such a balance with regard to race and ethnicity. |
| Regarding the statistical conclusions regarding the difference in slopes between White and Black participants, was this analysis determined before the data analysis started? Trying out different models may inflate the p-values, and I did not quite get the rationale behind the interaction described in line 169. | Thank you for your question regarding the statistical analysis. When we discovered that we needed a statistical model to adjust for sex and age, we decided on using linear mixed-effect models. We pre-specified only one model before fitting it. Therefore, we do not believe that the p-values are inflated.  Regarding the interaction term, thank you for pointing out that we need more clarification in the manuscript here. Without the interaction term between ratio and race in the model, the effect of ratio would have been the same for the two groups by design. We have revised the Methods section to clarify the role of the interaction term (line 185):  *“The linear mixed effect model approach was chosen because data points from the same individual cannot be considered independent measurements. A random intercept was included for individuals since it was assumed there would be variation in RMSE results between participants as the same individual contributed to the data with an RMSE for each ratio. The variance of the random intercepts describe between-person variability. The model included the following covariates: ratio (proportion of White and Black), race, age, sex, and training data sample size. An interaction term between race and ratio was added to model a potential divergence in performance by race because it was assumed training data composition affected performance. This means that a different slope was allowed for the two racial groups when the linear mixed effect models were fitted to the results so the hypothesis could be tested.”* |
| Line 94: “…study, 101 of whom were…” -> make new sentence, the connection with “whom” implies that 101 is a subset of 3. | Thank you for this suggestion. We have now revised the sentence to (line 107): “*We excluded three participants who did not complete the study, resulting in 205 participants included. Among them, 101 were non-Hispanic White (White), and the other 104 were non-Hispanic Black (Black).”* |
| Likewise, the formulation in line 122 is strange regarding the “11 training datasets assigned to them”. I would rather say that participants get assigned to training sets. | Please see our response to the second question. |
| Line 243: Provide a citation for the surveillance error grid. | Thank you for noticing the missing reference. It has now been added to the manuscript (line 261): “*Predictions from all models for all participants were evaluated with a surveillance error grid [24], …”*  *[24] Klonoff DC, Lias C, Vigersky R, Clarke W, Parkes JL, Sacks DB, et al. The Surveillance Error Grid. 2014; J Diabetes Sci Technol. 8: 658–672. doi:10.1177/1932296814539589* |

| **Reviewer 2** | Answers |
| --- | --- |
|  | We appreciate the feedback of the reviewer. Please find our point-by-point responses below. |
| While the abstract highlights a slope difference between non-Hispanic White and Black groups, the discussion section lacks an explanation or description of this finding. The authors should elaborate on how this observation affects the fairness of glucose predictions. | Thank you for pointing out the importance of placing more emphasis on fairness. The literature on algorithmic fairness in medicine almost exclusively focuses on binary outcomes. Therefore, our analysis is based on simple performance comparisons between Black and White populations and not well-established fairness metrics, which makes it more difficult to draw solid conclusions with regard to fairness. We have now elaborated more on the slope difference in the Discussion section (line 280):  *“We found that the racial composition of training data led to a small statistically significant divergence in performance between the slopes that were fitted to the results from White and Black participants when models were not personalized. This suggests increasing disparity, with performance differences favoring White individuals becoming more pronounced as their proportion in the dataset grows. Although the difference between the performances of the two racial groups might not be large enough to produce clinical inequalities in this study, the findings still indicate that the machine learning models could pick up on racial markers within the CGM data that was used in our study. This stresses the importance of training data composition from the perspective of algorithmic fairness to avoid increasing disparities between ethnic, racial, and demographic groups when considering integrating and using AI-driven healthcare tools. Performance differences disappeared when transfer learning was applied, demonstrating that the imbalance in training data can be mitigated with data from the underrepresented group in the initial training data”* |
| The study relies solely on the model for experimentation. Exploring additional machine learning models, such as traditional linear regression, multilayer perceptron, or advanced transformer-based models, could provide a broader understanding of potential racial disparities in healthcare AI applications. | Thank you for suggesting the exploration of different models, which we also considered when planning the study. While we acknowledge the value in testing and comparing different model architectures, we decided to prioritize clarity. The inclusion of multiple models would significantly increase the complexity of the Methods section and take the focus away from the racial comparison.  However, we conducted a thorough literature search on different methods when we started the project to make an informed decision about which model to use. Existing literature indicates a disagreement about which architectures perform best for blood glucose predictions. For example, van Doorn et al. [1] found LSTM to be effective, while Jaloli et al. [2] experimented with RNN, LSTM, and CNN-LSTM hybrids. Seo et al.[3] recommended a CNN architecture, and Lee et al. [4] suggested a transformer. However, all articles showed comparable performances. This suggests that the architecture is secondary to data quality. Given these considerations and the expertise of our team and co-authors, we chose the LSTM architecture.  [1] van Doorn WPTM, Foreman YD, Schaper NC, Savelberg HHCM, Koster A, van der Kallen CJH, et al. Machine learning-based glucose prediction with use of continuous glucose and physical activity monitoring data: The Maastricht Study. 2021; PLOS ONE. 16: e0253125. doi:10.1371/journal.pone.0253125  [2] Jaloli M, Cescon M. Long-Term Prediction of Blood Glucose Levels in Type 1 Diabetes Using a CNN-LSTM-Based Deep Neural Network. 2023; J Diabetes Sci Technol. 17: 1590–1601. doi:10.1177/19322968221092785  [3] Seo W, Park SW, Kim N, Jin SM, Park SM. A personalized blood glucose level prediction model with a fine-tuning strategy: A proof-of-concept study. 2021; Comput Methods Programs Biomed. 211: 106424. doi:10.1016/j.cmpb.2021.106424  [4] Lee Y-B, Kim G, Jun JE, Park H, Lee WJ, Hwang Y-C, et al. An Integrated Digital Health Care Platform for Diabetes Management With AI-Based Dietary Management: 48-Week Results From a Randomized Controlled Trial. 2023; Diabetes Care. 46: 959–966. doi:10.2337/dc22-1929 |
| The study's limitations acknowledge the exclusive focus on non-Hispanic White and Black participants but overlook other important demographic variables, such as age, Including these factors could enhance the scope of the research. | Thank you for bringing this to our attention and highlighting the importance of other sensitive attributes. We intentionally focused on racial disparities and, therefore, left out additional demographic variables to maintain clarity and focus in the study. The major challenge is the sample size of training sets because, in our design, the training sets can only be as large as the smallest subgroup, which is, in this case, 82 children. However, for the information of the reviewer, we conducted similar analyses comparing predictions among children and adults. Similar to the analysis by race, we also found divergence in performance (although borderline significant, p=0.053 for the base-generalized model) but not a significant effect of ratio within the groups. We believe that the clarity of the paper would suffer from the combination of the results already included in the manuscript. |
| The lack of external dataset evaluation and the relatively small sample size may limit the generalizability of the findings. The authors are encouraged to incorporate external datasets with varied racial compositions to assess racial disparities across datasets. | Thank you for your suggestions. While we acknowledge the value of incorporating an external dataset, unfortunately, we do not have access to any that includes both CGM data and a diverse racial composition. However, we believe that our work can inspire further research, incl. data collection and analysis in the field. We recognize this as a limitation in the manuscript (line 346):  “*Other groups, such as age strata, could also be relevant to investigate in the future as our study found that predicting blood glucose levels for children is more challenging than for adults. Another limitation of our study is that data was limited to the United States and might not generalize well to other countries as diabetes management and care practices vary globally. Furthermore, having another dataset for external evaluation and a larger sample size could have further strengthened the results. Lastly, data was collected between 2014 and 2017, and there have been improvements in CGM devices since, which could have improved data quality.”* |
